# Effect of Indoor Residual Spraying on Malaria in Pregnancy and Pregnancy Outcomes: A Systematic Review

**DOI:** 10.4269/ajtmh.24-0435

**Published:** 2024-10-29

**Authors:** Austin Oberlin, Tesia G. Kim, Adrienne Pettiette Erlinger, Avina Joshi, Halimatou Diawara, Sara A. Healy, Alassane Dicko, Patrick E. Duffy, Michele Hacker, Blair J. Wylie

**Affiliations:** ^1^ Department of Obstetrics and Gynecology, Columbia University Irving Medical Center, New York, New York;; ^2^ Department of Obstetrics and Gynecology, Mass General Brigham Medical Center, Boston, Massachusetts;; ^3^ Department of Obstetrics and Gynecology, Beth Israel Deaconness Medical Center, Boston, Massachusetts;; ^4^ Malaria Research and Training Centre, University of Science Techniques and Technologies of Bamako, Bamako, Mali;; ^5^ Laboratory of Malaria Immunology and Vaccinology, National Institute of Allergy and Infectious Diseases, National Institutes of Health, Bethesda, Maryland

## Abstract

Malaria in pregnancy increases maternal and perinatal morbidity and mortality. Indoor residual spraying (IRS) is a core vector control strategy used to reduce transmission in endemic areas; however, its efficacy in reducing the sequelae of malaria in pregnancy is not well described. PubMed, Embase, Cochrane, and Web of Science were searched for all studies assessing IRS exposure during pregnancy. Abstracts and full texts were reviewed independently by two researchers, with discrepancies adjudicated by a third. Of 3,319 studies that met the search criteria, 17 met the inclusion criteria. Thirteen studies reported on the effect of IRS on malaria endpoints during pregnancy, five on birth outcomes, and one on a fetal anomaly. Twelve of the 13 studies exploring maternal malaria and 3 of 3 studies reporting on placental malaria demonstrated a reduction among those exposed to IRS during pregnancy. Results were more mixed for obstetric outcomes. Two of the best-quality studies showed reductions in preterm birth, low birthweight, and fetal/neonatal mortality; a third high-quality study did not demonstrate a reduction in perinatal mortality but did not evaluate preterm birth. One study found a significantly increased risk of preterm birth in those exposed to IRS, although the study was of lower quality. A final study demonstrated a small, although statistically significant, association between IRS and male urogenital birth defects. In malaria-endemic areas, the published literature suggests that IRS during pregnancy reduces the incidence of malaria parasitemia. However, without high-quality prospective studies directly examining IRS in pregnancy, the impact on birth outcomes is less clear.

## INTRODUCTION

Despite decades of efforts at eradication, malaria remains an ongoing threat to over half the world’s population and is a leading cause of morbidity and mortality for those in sub-Saharan Africa. In 2021, 96% of the world’s estimated 619,000 deaths from malaria occurred in this region.^1^ Pregnancy is an especially vulnerable time, as pregnant women are at increased risk of severe malaria, miscarriage or stillbirth, preterm birth (PTB), and delivering a child with low birthweight (LBW).^2^^–^^4^ Given this, the WHO recommends several strategies for malaria prevention in pregnancy: sleeping under insecticide-treated nets/long-lasting insecticide nets (ITNs/LLINs), indoor residual spraying (IRS) of insecticides, and intermittent preventive treatment of malaria in pregnancy (IPTp) with sulfadoxine-pyrimethamine.^5^ The proportion of pregnant women sleeping under ITNs in endemic areas continues to grow, with 53% of pregnant women owning ITNs as of 2021. Similarly, 55% of pregnant women in the WHO Africa region received at least one dose of IPTp (of the three or more recommended doses). Conversely, the proportion of those at risk, including pregnant persons and children, who are protected by IRS has fallen to only 2.4%.^1^

Indoor residual spraying refers to the spraying of a long-lasting insecticide on the interior surfaces of all houses or other structures in an endemic area where vectors (i.e., mosquitoes) might land.^6^ Unlike other malaria prevention strategies, such as the use of ITNs, IRS works to decrease the overall vector population in a community. Additionally, most insecticides last for 6 months or longer and do not require individual users to change behavior for them to be effective. However, IRS is most effective in coordinated campaigns. Typically, at least 85% of households in an area must be sprayed to see reduced malaria transmission among the population.^5^ A variety of insecticides are used for IRS across four classes: organochlorines, pyrethroids, organophosphates, and carbamates.^7^ There is no consensus opinion on the safety of their use in IRS around pregnant persons.

Several systematic reviews have been performed to summarize the efficacy of IRS on malaria prevention, although none focus on efficacy in the pregnant population. Most recently, Zhou et al. reported on 38 studies exploring malaria outcomes and IRS, of which 19 were exclusively in children. Overall, IRS reduces malaria prevalence by approximately 60%, although much heterogeneity exists across studies.^8^^,^^9^ Although it is anticipated that parasitemia will be reduced among pregnant people exposed to IRS, the impact on birth outcomes may be less predictable as prenatal exposures to some insecticides in nonendemic areas has been associated with decrements in birth size and increased risk of PTB.^10^^–^^12^

Prevention of malaria in pregnancy has been shown to reduce maternal anemia, the risk of LBW, and the incidence of perinatal mortality.^13^ Before ministries of health or international health organizations make decisions on whether to continue, increase, or abandon investment in IRS, it is essential that they weigh the potential impact on pregnancy outcomes in the risk–benefit calculus. This systematic review was conducted to answer the following questions: does exposure to IRS during pregnancy alter the incidence of maternal or placental parasitemia or maternal anemia, and does exposure to IRS affect birth outcomes?

## MATERIALS AND METHODS

This systematic review was conducted following the Preferred Reporting Items for Systematic Reviews and Meta-Analyses (PRISMA) guidelines.^14^

### Search methods for identification of studies.

A systematic review of published literature from PubMed, Embase, Cochrane, and Web of Science was conducted with the assistance of a research librarian. The initial search was conducted in March 2020 and updated in January 2024. No date limitations were considered, and only manuscripts available in English were included. The review was registered with the International Prospective Register of Systematic Reviews (CRD42020162049) and the protocol is published online (https://www.crd.york.ac.uk/prospero/display_record.php?RecordID=162049). The PRISMA statement and detailed search terms are available in Supplemental Appendix 1.^14^ Included in the analysis were interventional and observational studies where IRS was evaluated as a predictor of pregnancy outcomes, maternal malaria, or maternal/placental parasitemia. Editorials or reviews without original data, ongoing clinical trials, or abstract presentations at conferences were excluded. Exposure to IRS was defined as exposure to insecticide through coordinated spraying programs (i.e., government, nongovernmental organization, or research programs). Studies that reported on serum or urinary insecticide biomarker concentrations without reference to whether there was exposure to an IRS program were excluded. Additionally, in cases where the exposure was personal insecticide sprays rather than systematically performed IRS, these were excluded from our analysis but are summarized in Supplemental Appendix 3. Studies were not excluded based on any other aspects of their patient population or other interventions used in the prevention of malaria.

### Data collection and analysis.

**Figure 1.**
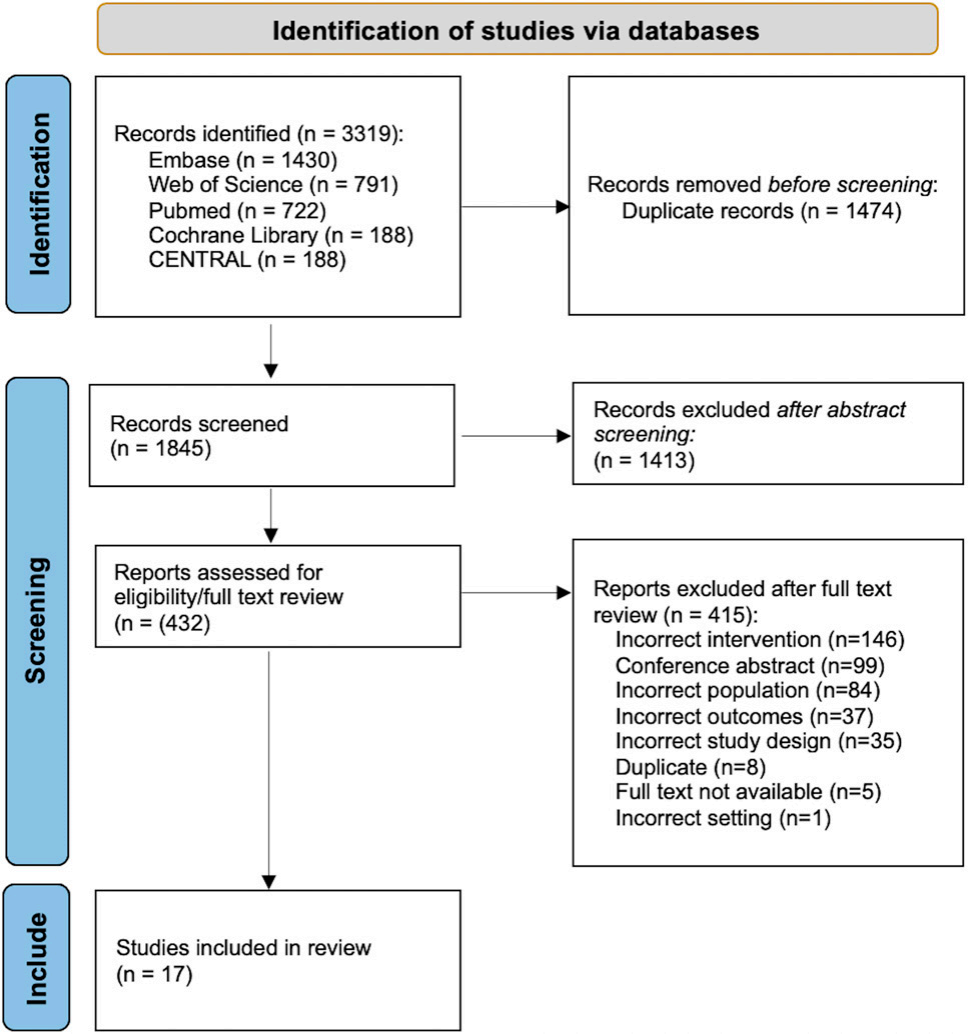
Preferred Reporting Items for Systematic Reviews and Meta-Analyses flow diagram of included studies.

All studies meeting the search criteria were uploaded into Covidence, a systematic review management software (Veritas Health Innovation, Ltd., Melbourne, Australia). Two independent researchers screened all abstracts for full-text review. Full texts were then independently reviewed by the two researchers for inclusion. Discrepancies for inclusion of abstracts and full texts were resolved by a third reviewer. The study selection process is illustrated in Figure 1.

### Data extraction and management.

An extraction tool was created within Covidence, and all data were abstracted and recorded within this software. A summary of the included study characteristics is included in Supplemental Appendix 4. As described above, these characteristics were abstracted by two independent reviewers, and any discrepancies were resolved by a third reviewer. Abstracted characteristics include primary study aim, design, dates of data collection, number of participants, inclusion and exclusion criteria for participants, study location and its malaria endemicity, how IRS exposure was defined and any details about the insecticide used, definitions of included outcomes, and any special notes made by the reviewers.

Data were abstracted for all outcomes in which IRS was considered an exposure. These included maternal peripheral parasitemia or clinical malaria, placental parasitemia or placental malaria by histopathology, maternal anemia, PTB, LBW, fetal/neonatal mortality, and neonatal anomalies. Maternal parasitemia was defined as a binary outcome assessed via a variety of methods, including microscopy (blood smear), rapid diagnostic test (RDT), loop-mediated isothermal amplification (LAMP), or nucleic acid amplification via PCR. Clinical malaria was a binary outcome defined as signs or symptoms of malaria that were either identified by a clinician or self-reported, without confirming parasitemia. Similarly placental malaria was a binary outcome that could be determined with a variety of methods, including microscopic examination, LAMP, PCR, or histopathology. When more than one method was used in a given study, histopathology was reported, as the gold-standard for diagnosis, and a note was made about the outcomes of other methods. PTB was defined as delivery at less than 37 weeks gestation and was considered a binary outcome. LBW was a binary outcome defined as a newborn birthweight of less than 2,500 g. If a study reported on gestational age at delivery or birthweight as a continuous variable, this was recorded separately. Fetal/neonatal mortality was a binary outcome including any pregnancy that resulted in a stillbirth/intrauterine fetal demise (as defined by study) or neonatal death (demise at less than 28 days of life). Finally, any neonatal anomalies were reported as defined by the included study.

When a study reported on different levels of the exposure (e.g., direct versus indirect spraying), all data were abstracted and included. When available, both crude and adjusted effect sizes were reported. In some cases, the crude effect size was calculated from available data. To ease comparison across studies, all crude effect sizes were reported as IRS exposed versus unexposed. In cases where we inverted the relationship reported in the primary literature, a footnote was made. Because adjusted effect sizes could not be reoriented in this way, we have made note in the tables when the relationship is different. In any case where data could not be found in the primary manuscript or supplemental material, it is noted as not reported.

To ease comparison across studies, forest plots were created for any outcome reported by at least four studies. The measure of association and 95% CI from the original study were reported. As these differed across studies, a footnote is included below the figure. When possible, the adjusted effect size was included. However, in cases where the association was inverted (i.e., unexposed versus exposed), the crude ratio was used to ease comparison across studies. Both the crude and adjusted effect sizes are additionally listed in the tables for further reference. An overall effect size is not included in the forest plots as a meta-analysis was not performed.

### Assessment of risk of bias in included studies.

After full-text review, studies that met the inclusion criteria were either observational cohort or cross-sectional studies. Although three studies were secondary analyses of randomized controlled trials, the primary interventions were not IRS, and so these were functionally prospective cohorts. The NIH Quality Assessment Tool for Observational Cohort and Cross-Sectional Studies was used by two independent reviewers. Studies were evaluated on a “Good, Fair, or Poor” scale for the clarity of the objectives and study populations, participation rates and recruitment (i.e., <20% lost to follow-up), statistical strength (sample size and power), and adequate study period time to assess outcomes. The reviewers assessed study criteria based on the research question asked in this review. In cases where a study’s primary objective was different than our stated research question, this may be reflected in our assessment of the quality of the study for purposes of this review.

## RESULTS

### Study selection.

A total of 3,319 records met the electronic search criteria and were imported into the Covidence software. Before abstract screening, 1,474 duplicate records were excluded. Subsequently, 1,845 abstracts were screened, of which 1,413 were excluded based on the criteria detailed above, and 432 were advanced for full-text review (Figure 1). Of these, 17 were identified for inclusion in the review, whereas 415 were excluded. The most common reasons for exclusion were an intervention other than IRS, study population other than pregnant persons, or outcomes other than those defined above. Of note, six studies were excluded as incorrect interventions because, on detailed review, the exposure was an “insecticide spray” with a lack of clarity as to whether they were deployed by an individual household or as a component of community IRS campaigns. These six studies are included in an appendix for review by interested readers (Supplemental Appendix 3).^15^^–^^20^ Several studies did not meet the criteria for a variety of other reasons, including conference abstracts or incorrect study design or the full text could not be found. Finally, one study was excluded as it was set in a high-income country where malaria is not endemic.^21^

### Included studies.

Characteristics of the studies that were included can be found in the Supplemental Appendix 4, and a brief summary is provided in Table 1. A total of 17 studies met the inclusion criteria. One study, Roh et al.,^22^ was a quasiexperimental retrospective study. Three were prospective cohort studies (Muhindo et al.,^23^ Roh et al.,^24^ and Uwimana et al.^25^), which evaluated the effect of IRS as part of a secondary analysis of randomized controlled trials assessing a different intervention. The remaining 13 studies were cross-sectional (Hamer et al.,^26^ Bornman et al.,^27^ Lee et al.,^28^ Lee et al.,^29^ Tongo et al.,^30^ Nega et al.,^31^ Kamuliwo et al.,^32^ Tilahun et al.,^33^ Subussa et al.,^34^ Alhassan et al.,^35^ Gemechu et al.,^36^ Balcha et al.,^37^ and Eboumbou Moukoko et al.^38^). Included studies were published between 2009 and 2023 across 10 countries (Cameroon, Ethiopia, Ghana, India, Nigeria, Rwanda, Sao Tome and Principe, South Africa, Uganda, and Zambia). No studies published before 2009 were identified that met inclusion criteria.

**Table 1:** Summary of included studies

Study	Study Design	Population	Location	Malaria Incidence in Study Area (per 1,000)*	Quality Assessment
Hamer et al.^26^	Cross-sectional	3,104 Pregnant persons enrolled at antenatal clinics or delivery hospitals	Jharkhand State, India	3	Fair
Bornman et al.^27^	Cross-sectional	3,310 Pregnant person–newborn dyads enrolled at time of delivery in provincial hospital	Limpopo, South Africa	0.5	Good
Lee et al.^28^	Cross-sectional	Undefined number of pregnant persons, data from a nationwide governmental database	Principe	12	Poor
Lee et al.^29^	Cross-sectional	17,851 Pregnant persons from a nationwide government database	Sao Tome and Principe	12	Poor
Tongo et al.^30^	Cross-sectional	796 Pregnant persons enrolled at time of delivery in two urban hospitals	Ibadan, Nigeria	307	Fair
Nega et al.^31^	Cross-sectional	341 Pregnant persons randomly sampled from the community during pregnancy	Arbaminch Town, Ethiopia	46	Fair
Kamuliwo et al.^32^	Cross-sectional	Undefined number of pregnant persons, data from a nationwide governmental database	Zambia	188	Fair
Muhindo et al.^23^	Prospective cohort constructed within RCT	289 HIV-uninfected pregnant persons recruited between 12 and 20 weeks and followed through delivery	Tororo, Uganda	284	Good
Roh et al.^24^	Prospective cohort constructed within two different RCTs	565 Pregnant persons living with HIV recruited between 12 and 28 weeks and followed through delivery	Tororo, Uganda	284	Good
Tilahun et al.^33^	Cross-sectional	331 Pregnant persons recruited from two antenatal care clinics with convenience sampling	Jawi District, Ethiopia	46	Fair
Subussa et al.^34^	Cross-sectional	364 Pregnant persons recruited from randomly selected geographic area within a rural district	Merti district, Oromia, Ethiopia	46	Fair
Alhassan et al.^35^	Cross-sectional	350 Pregnant persons, data from nationwide Ghana Malaria Indicator Survey	Ghana	164	Fair
Roh et al.^22^	Retrospective quasiexperimental study	59,992 Delivery records, data from randomly selected health facilities from nationwide government database	Eastern Uganda	284	Good
Uwimana et al.^25^	Prospective cohort within a cluster RCT	1,786 Pregnant persons recruited consecutively at first antenatal care visit and followed through delivery	Kamonyi and Huye districts, Southern Province, Rwanda	126	Good
Gemechu et al.^36^	Cross-sectional	557 Pregnant persons recruited from randomly selected geographic area within a rural district	West Guji Zone, Oromia, Ethiopia	46	Fair
Balcha et al.^37^	Cross-sectional	328 Pregnant persons recruited from the community within a rural district	Boset District, Oromia, Ethiopia	46	Fair
Eboumbou Moukoko et al.^38^	Cross-sectional	888 Pregnant persons recruited from antenatal clinics associated with two hospitals	Littoral region, Cameroon	245	Fair

RCT = randomized controlled trial.

* WHO, Global Health Observatory Data Repository/World Health Statistics (apps.who.int/gho/data).

Of the 17 studies included, 5 had a primary aim of understanding the effect of IRS on pregnancy outcomes (Bornman et al.,^27^ Muhindo et al.,^23^ Roh et al.,^24^ Alhassan et al.,^35^ and Roh et al.^22^). Bornman et al.^27^ aimed to determine the association between IRS exposure and neonatal urogenital malformation. This study did not evaluate or control for other malaria prevention strategies. Muhindo et al.^23^ and Roh et al.^24^ reported IRS as a secondary outcome of prospective randomized trials, whose primary aims were to study IPTp and HIV in pregnancy, respectively. Both collected data on ITN/LLIN and IPTp use and controlled for these in adjusted effect estimates of IRS. Alhassan et al.^35^ reported on both IRS and ITN use as primary intervention strategies to prevent malaria but did not control for these in an adjusted model. Finally, Roh et al.^22^ performed a quasiexperimental analysis primarily examining the effect of IRS exposure on pregnancy outcomes. In this difference-in-differences methodology, ITN and IPTp use were not reported, and estimates could have been subject to unmeasured confounding had these trends differed between IRS and control groups over time.

Of the remaining 12 studies, 11 were cross-sectional studies where the primary aim was to explore a variety of malaria prevention strategies in pregnancy (Table 1). These 11 cross-sectional studies report on a wide variety of malaria prevention strategies, and 7 studies controlled for these in adjusted models. The one prospective study, Uwimana et al., examined IRS exposure as a secondary exposure within a prospective randomized trial. The primary intervention in the trial was intermittent screening and treatment of malaria in pregnancy, and an adjusted effect estimate was performed for IRS.^25^

Thirteen of the studies evaluated maternal malaria, of which 11 reported asymptomatic cases of peripheral parasitemia,^22^^,^^23^^,^^26^^,^^28^^,^^29^^,^^31^^,^^33^^,^^34^^,^^36^^–^^38^ 1 reported symptomatic parasitemia,^32^ and 1 self-reported clinical malaria.^35^ One study reported separately on maternal anemia in relation to exposure to IRS.^25^ Additionally, three studies evaluated placental malaria.^22^^,^^23^^,^^25^ Five studies reported an obstetric outcome, including four studies documenting the association of IRS with PTB,^22^^,^^23^^,^^25^^,^^30^ five reporting an association with LBW,^22^^–^^25^^,^^30^ and three reporting on fetal/neonatal mortality.^22^^–^^24^ Finally, one study reported on a newborn anomaly, specifically external urogenital birth defects.^27^

### IRS exposure.

Nine of the 17 studies defined IRS exposure as present if the participant reported having their home sprayed within the past 12 months.^26^^,^^30^^,^^31^^,^^33^^–^^38^ Eight studies used the approximate date, based on government records that an IRS campaign was performed to define whether a pregnancy was exposed to IRS.^22^^–^^25^^,^^27^^–^^29^^,^^32^ Only two studies, Muhindo et al.^23^ and Roh et al.,^24^ reported on the proportion of a pregnancy considered protected by IRS by comparing the participant’s date of IRS spray with their estimated due date. These two studies then divided the population into two levels of coverage across gestation, although the defined levels were different between the two studies. Muhindo et al.^23^ compared unexposed participants with those exposed “>0% to 20%” and to those exposed “>20 to 43%” because the longest that participants in their cohort were exposed was 43% of the pregnancy. Roh et al.^24^ were able to compare unexposed participants to those with “>0% to 90%” of the pregnancy exposed to IRS and to those with “>90%” of the pregnancy exposed as their cohort had longer exposure to IRS overall. Additionally, Muhindo et al.^23^ reported on whether a pregnant person’s household was directly sprayed or whether the surrounding homes were sprayed and compared these groups to those unexposed to IRS. All other studies treated participants as “IRS exposed” if they self-reported exposure or if government records indicated spraying of their village or district.

Nine of the 17 included studies did not report on the specific insecticide used.^30^^–^^38^ Of the remaining eight studies, two studies reported use of pyrethroid insecticides (Lee et al.^28^ and Lee et al.^29^), three studies reported use of carbamate insecticides (Muhindo et al.,^23^ Roh et al.,^24^ and Uwimana et al.^25^), one study reported use of dichlorodiphenyltrichloroethane (DDT; Bornman et al.^27^), one study used a combination of DDT and pyrethroids (Hamer et al.^26^), and one study used a combination of carbamate and organophosphates (Roh et al.^22^).

### Outcomes.

#### Malaria outcomes.

**Figure 2.**
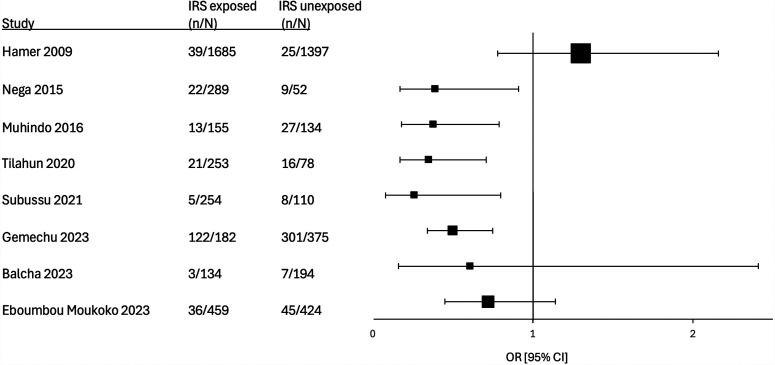
Forest plot of studies comparing maternal peripheral parasitemia between those exposed and those not exposed to indoor residual spraying (IRS). In Hamer et al.^26^ and Muhindo et al.,^23^ the two subgroups are combined to calculate a single odds ratio (OR).^ †^Study used an unadjusted OR; ^‡^study used adjusted OR; and ^#^study used adjusted incidence RR.

Eleven studies evaluated the association between IRS exposure and asymptomatic maternal peripheral parasitemia. In all but one of these studies, the incidence of parasitemia was lower among those exposed to IRS (Figure 2).^22^^,^^24^^,^^28^^,^^29^^,^^31^^,^^33^^,^^34^^,^^36^^–^^38^ In the two prospective studies that reported an adjusted effect estimate, the findings showed significantly lower incidence of peripheral maternal parasitemia in those exposed to IRS (Muhindo et al.^23^: adjusted RR [aRR]: 0.42 [95% CI 0.05–0.61], Roh et al.^24^: adjusted incidence RR [IRR] 0.07 [95% CI 0.009–0.48]). One study (Kamuliwo et al.) evaluated the effect of IRS on symptomatic malaria as diagnosed by peripheral parasitemia. They found a small reduction in those exposed to IRS (prevalence RR: 0.98 [95% CI 0.98–0.99]).^32^ Finally, Alhassan et al.^35^ conclude that there was a significant reduction in self-reported malaria among those with exposure to IRS (average treatment effect: –7.36% [95% CI –13.45–1.27%]), although only 1.2% of the population reported exposure to IRS. One study (Uwimana et al. reported the impact of IRS on maternal anemia as an outcome. Uwimana et al.^25^ found a 57% reduction in moderate to severe maternal anemia at delivery (aRR of maternal anemia <10 mg/dL: 0.43 [95% CI 0.18–1.02]).

Three studies (Muhindo et al.,^23^ Roh et al.,^24^ and Uwimana et al.^25^) reported on the incidence of placental malaria. All three studies showed a lower risk of placental malaria in those exposed to IRS, and this remained significant in two of the three studies after adjusting for confounders (Roh et al. 2017: aRR by histopathology: 0.20 [95% CI 0.08–0.47], Uwimana et al.: aRR by PCR: 0.33 [95% CI 0.23–0.48]; Table 2).^23^^–^^25^ Additionally, in both Muhindo et al.^23^ and Roh et al.,^24^ the reduction was evident regardless of the length of pregnancy protected by IRS, although not in a dose-dependent fashion.

**Table 2:** Summary of studies comparing maternal and placental malaria outcomes with exposure versus no exposure to indoor residual spraying

Study	Sample Size/Details *N*	IRS Exposed *n* (%)	IRS Unexposed *n* (%)	Effect Size IRS Exposed vs. Unexposed Crude Ratio (95% CI)	Effect Size*, Adjusted (95% CI)
Peripheral Parasitemia (asymptomatic)					
Hamer et al.^26^	3,104 Pregnant women, asymptomatic recruited antepartum or at delivery	1,688 (54.4) *Enrolled antepartum*: 1,270/2,386 (53.5)	1,416 (45.6) *Enrolled antepartum*: 1,116/2,386 (46.8)	*Enrolled antepartum*: 1.8% vs. 1.8% RR: 1.00 (0.55–1.81)	Not reported
		*Enrolled at delivery*: 418/718 (58.5)	*Enrolled at delivery*: 300/718 (41.8)	*Enrolled at delivery*: 3.9% vs. 1.7% RR: 2.26 (0.84–6.10)	Not reported
Lee et al.^28^	Undefined number of pregnant persons, data from a nationwide governmental database	Not reported	Not reported	Incidence of malaria: −96% between 2003 and 2009 (pre- and post-IRS)	Not reported
Lee et al.^29^	17,851 Pregnant persons from a nationwide government database	Not reported	Not reported	Incidence of malaria: 30.8% in 2004 to 1.3% in 2009 (pre- and post-IRS)	Not reported
Nega et al.^31^	341 Pregnant women, asymptomatic	289 (84.8)	52 (15.2)	7.6% vs. 17.3% OR*: 0.39 (0.17–0.91)	*Unexposed vs. exposed* *aOR* ^†^ *: 2.19 (0.6–7.76)*
Muhindo et al.^23^	289 Pregnant women, asymptomatic at study intake	155 (53.6) *Individuals whose homes directly sprayed:* 137	134 (46.3)	*Directly sprayed* 8.5% vs. 20.2% OR: 0.38 (0.18–0.79)	*Directly sprayed* *exposed vs. unexposed* aRR^‡^: 0.42 (0.05–0.61)
		*Individuals where surrounding homes sprayed (indirect community exposure):* 18		*Indirect community exposure* 5.0% vs. 34.2% OR: 0.23 (0.03–1.83)	*Indirect community exposure* *exposed vs. unexposed* aRR^‡^: 0.12 (0.03–0.59)
Roh et al.^24^	565 Pregnant women living with HIV, asymptomatic at study intake	185 (32.7)	380 (67.3)	IRR: 0.03 (0.004–0.21)	*Exposed vs. unexposed* aIRR^§^ 0.07 (0.009–0.48)
Tilahun et al.^33^	331 Pregnant women, asymptomatic	253 (76.4)	78 (23.6)	8.3% vs. 20.5% OR*: 0.35 (0.17–0.71)	*Unexposed vs. exposed* *aOR* ^‖^ *: 3.13 (1.47–6.66)*
Subussa et al.^34^	364 Pregnant women, asymptomatic	254 (69.8)	110 (30.2)	2.0% vs. 7.2% OR*: 0.26 (0.08–0.80)	*Unexposed vs. exposed* *aOR* ^¶^ *: 1.75 (0.44–6.57)*
Gemechu et al.^36^	557 Pregnant women, asymptomatic	182 (32.7)	375 (67.3)	19.7% vs. 33.0% OR*: 0.50 (0.34–0.75)	*Unexposed vs. exposed* *aOR: 1.9 (1.27–3.05)*
Balcha et al.^37^	328 Pregnant women, asymptomatic	134 (40.9)	194 (59.1)	2.2% vs. 3.6% OR*: 0.61 (0.16–2.41)	*Unexposed vs. exposed* aOR: 2.70 (0.65–11.17)
Eboumbou Moukoko et al.^38^	883 Pregnant women, asymptomatic	459 (52.0)	424 (48.0)	7.8% vs. 10.6% OR: 0.72 (0.45–1.14)	*Exposed vs. unexposed* aOR: 0.53 (0.30–0.95)
Peripheral Parasitemia (symptomatic)					
Kamuliwo et al.^32^	398,104 Cases of malaria in pregnancy reported in a national database	Not reported	Not reported	Prevalence RR: 0.98 (0.98–0.99)	Not reported
Self-Reported Malaria					
Alhassan et al.^35^	350 Pregnant women	4 (1.2)	346 (98.8)	ATE^#^: –7.36% (–13.45%, 1.27%) *P* = 0.018	Not reported
Maternal Anemia					
Uwimana et al.^25^	1,688 Pregnant women	746 (44.2)	942 (55.8)	*Moderate to severe maternal anemia at delivery (Hb <10 mg/dL)* RR: 0.39 (0.15–1.05)	*Exposed vs. unexposed* aRR**: 0.43 (0.18–1.02)
Placental Malaria					
Muhindo et al.^23^^††^	289 Pregnant women	155 (53.6) *>0–20% exposure in pregnancy:* 90 (31.1)	134 (46.3)	*Exposed (>0–20%) vs. unexposed* 28.4% vs. 47.7% OR: 0.43 (0.24–0.77)	*Exposed (>0–20%) vs. unexposed* aOR: 0.77 (0.35–1.69)
		*>20–43% exposure in pregnancy:* 65 (22.5)		*Exposed (>20–43%) vs. unexposed* 27.4% vs. 47.7% OR: 0.41 (0.22–0.80)	*Exposed (>20–43%) vs. unexposed* aOR: 0.63 (0.27–1.48)
Roh et al.^24^^††^	565 Pregnant women living with HIV	185 (32.7) *>0–90% of pregnancy:* 88 (15.6)	380 (67.3)	*Exposed (>0–90%) vs. unexposed (0%)* RR**: 0.12 (0.04–0.37)	*Exposed (>0–90%) vs. unexposed (0%)* aRR: 0.12 (0.04–0.36)
		*>90% of pregnancy:* 97 (17.2)		*Exposed (>90%) vs. unexposed (0%)* RR: 0.19 (0.08–0.44)	*Exposed (>90%) vs. unexposed (0%)* aRR^‡‡^: 0.20 (0.08–0.47)
Uwimana et al.^25^	1,688 Pregnant women	746 (44.2)	942 (55.8)	RR^‡‡^: 0.31 (0.21–0.47)	*Exposed vs. unexposed* aRR^‡‡^: 0.33 (0.23 – 0.48)

aOR = adjusted odds ratio; aRR = adjusted RR; ATE = average treatment effect; Hb = hemoglobin; IRR = incidence RR; IRS = indoor residual spraying.

Adjusted effect sizes are reported as in the original manuscripts with notations as to whether the referent was unexposed or exposed. When exposure was the referent (unexposed vs. exposed), the results are italicized.

* Ratio is the inverse of that reported in the original manuscript to keep associations in the same direction for unadjusted effect size (exposed vs. unexposed).

^†^ Nega et al.^31^ adjusted for gravidity, insecticide-treated net (ITN) use, and age.

^‡^ Muhindo et al.^23^ adjusted for gravidity, age, use of intermittent preventive treatment (IPT), and gestational age when IPT started.

^§^ Roh et al.^24^ adjusted for education, household wealth, gravidity, use of IPT, and age.

^‖^ Tilahun et al.^33^ adjusted for residence, age, and gravidity.

^¶^ Subussa et al.^34^ adjusted for residence, age, gestational age, previous infection with *Plasmodium*, ITN use, and ANC attendance.

^#^ Average treatment effect is the absolute reduction in self-reported malaria prevalence, as estimated by a Poisson regression model.

** Uwimana et al.^25^ adjusted for study arm, gravidity, and baseline Hb level.

^††^ Muhindo et al.^23^ and Roh et al.^24^ rates and effect sizes are for histopathology. Both studies also reported microscopy and loop-mediated isothermal amplification/PCR as measures of placental malaria, which demonstrated a similar effect size and direction as the gold standard of histopathology.

^‡‡^ Uwimana et al.^25^ reported placental malaria by PCR only. Effect size is adjusted for study arm, gravidity, fever during pregnancy (at least one episode), and total number of antenatal care visits.

#### Pregnancy and neonatal outcomes.

**Figure 3.**
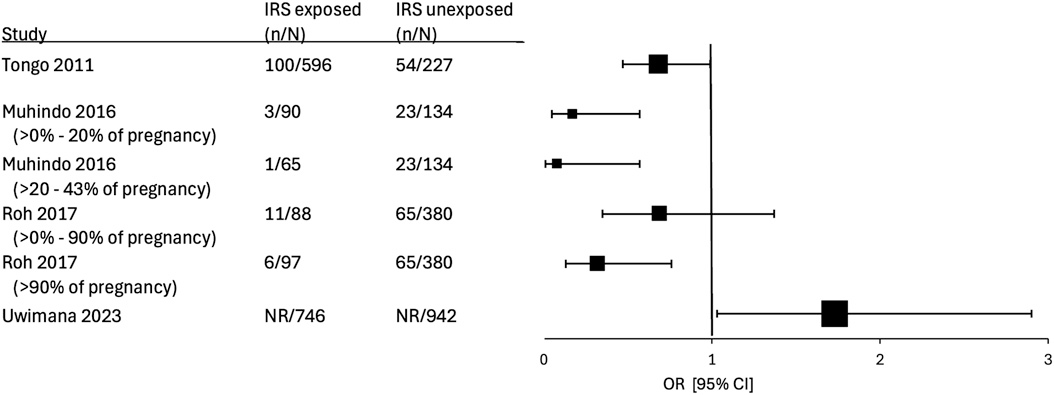
Forest plot of studies comparing preterm birth between those exposed and those not exposed to indoor residual spraying (IRS). ^†^Study used an unadjusted odds ratio (OR); ^†^study used adjusted OR; and ^&^study used adjusted RR.

Five studies reported on the effects of IRS on obstetric outcomes (PTB: four studies; LBW: five studies; fetal/neonatal mortality: three studies), and one reported a newborn anomaly (Table 3). Muhindo et al.,^23^ reported a reduction in PTB, which was greater the longer a pregnancy was protected by IRS (aRR of 0.13 [95% CI 0.03–0.53] in those exposed >0–20% of pregnancy compared with an aRR of 0.05 [95% CI 0.01–0.43] if pregnancy exposed >20–43%; Figure 3). Roh et al.,^24^ showed a similar reduction in PTB, with a larger magnitude of reduction the longer a pregnancy was exposed. However, this reduction was only statistically significant in the group exposed for at least 90% of the pregnancy (aRR: 0.35 [95% CI 0.15–0.84]). Conversely, Uwimana et al.,^25^ showed a significantly higher rate of PTB in those exposed to IRS (aRR: 1.76 [95% CI 1.03–3.02]).

**Table 3:** Summary of studies reporting obstetric and neonatal outcomes among those exposed to indoor residual spraying

Study	Sample Size/Details *N*	IRS Exposed *n* (%)	IRS Unexposed *n* (%)	Effect Size IRS Exposed vs. Unexposed Crude Ratio (95% CI)	Effect Size Adjusted Ratio (95% CI)
Preterm Birth (<37 weeks)					
Tongo et al^30^	796 Pregnant women at delivery	569 (71.5)	227 (28.5)	17.6% vs. 23.8% OR*: 0.68 (0.47–0.99)	*Unexposed vs. exposed* aOR^†^: 1.71 (1.15–2.56)
Muhindo et al.^23^	289 Pregnant women	155 (53.6) *>0–20% of pregnancy:* 90 (31.1)	134 (46.3)	*Exposed (>0–20%) vs. unexposed (0%)* 3.3% vs. 17.2% OR: 0.17 (0.05–0.57)	*Exposed (>0–20%) vs. unexposed (0%)* aOR^‡^: 0.13 (0.03–0.53)
		*>20–43% of pregnancy:* 65 (22.5)		*Exposed (>20–43%) vs. unexposed (0%)* 1.5% vs. 17.2% OR: 0.08 (0.01–0.57)	*Exposed (>20–43%) vs. unexposed (0%)* aOR^‡^: 0.05 (0.01–0.43)
Roh et al.^24^	565 Pregnant women living with HIV	185 (32.7) *>0–90% of pregnancy:* 88 (15.6)	380 (67.3)	*Exposed (>0–90%) vs. unexposed (0%)* 12.5% vs. 17.1% RR: 0.73 (0.40–1.33)	*Exposed (>0–90%) vs. unexposed (0%)* aRR^§^: 0.76 (0.35–1.65)
		*>90% of pregnancy:* 97 (17.2)		*Exposed (>90%) vs. unexposed (0%)* 6.2% vs. 17.1% RR: 0.36 (0.16–0.81)	*Exposed (>90%) vs. unexposed (0%)* aRR^§^: 0.35 (0.15–0.84)
Uwimana et al.^25^	1,688 Pregnant women	746 (44.2)	942 (55.8)	Rates not reported RR: 1.73 (1.03–2.9)	*Exposed vs. unexposed* aRR: 1.76 (1.03–3.02)
Low Birth Weight (<2,500 g)					
Tongo et al.^30^	796 Pregnant women at delivery	569 (71.5)	227 (28.5)	7.9% vs. 11.9% OR*: 0.64 (0.38–1.05)	*Unexposed vs. exposed* aOR^†^: 1.55 (0.90–2.67)
Muhindo et al.^23^	289 Pregnant women	155 (53.6) *>0–20% of pregnancy:* 90 (31.1)	134 (46.3)	*Exposed (>0–20%) vs. unexposed (0%)* 7.9% vs. 18.8% OR: 0.37 (0.15–0.89)	*Exposed (>0–20%) vs. unexposed (0%)* aOR^‡^: 0.29 (0.12–0.75)
		*>20–43% of pregnancy:* 65 (22.5)		*Exposed (>20–43%) vs. unexposed (0%)* 2.2% vs. 18.8% OR: 0.07 (0.01–0.51)	*Exposed (>20–43%) vs. unexposed (0%)* aOR^‡^: 0.08 (0.02–0.39)
Roh et al.^24^	565 Pregnant women living with HIV	185 (32.7) *>0–90% of pregnancy:* 88 (15.6)	380 (67.3)	*Exposed (>0–90%) vs. unexposed (0%)* 17.1% vs. 18.5% RR: 0.92 (0.55–1.53)	*Exposed (>0–90%) vs. unexposed (0%)* aRR^§^: 1.27 (0.69–2.36)
		*>90% of pregnancy:* 97 (17.2)		*Exposed (>90%) vs. unexposed (0%)* 9.3% vs. 18.5% RR: 0.50 (0.26 – 0.97)	*Exposed (>90%) vs. unexposed (0%)* aRR^§^: 0.68 (0.29–1.57)
Roh et al.^22^	59,992 Singleton deliveries	16,800 (28.0)	43,192 (72.0)	Not reported	*Exposed vs. unexposed* overall IRR^‖^: 0.67 (0.49–0.93) first year after IRS start IRR: 0.72 (0.50–1.03) second year after IRS start IRR: 0.62 (0.42–0.92)
Uwimana et al.^25^	1,688 Pregnant women	746 (44.2)	942 (55.8)	Rates not reported RR: 0.92 (0.63–1.34)	Exposed vs. unexposed aRR^¶^: 1.04 (0.69–1.56)
Fetal/Neonatal Mortality (stillbirth/neonatal demise under 28 days)^#^					
Muhindo et al.^23^	289 Pregnant women	155 (53.6) *>0–20% of pregnancy:* 90 (31.1)	134 (46.3)	*Exposed (>0–20%) vs. unexposed (0%)* 1.1% vs. 7.5% OR: 0.14 (0.02–1.11)	*Exposed (>0–20%) vs. unexposed (0%)* aOR^‡^: 0.10 (0.0–0.86)
		*>20–43% of pregnancy:* 65 (22.5)		*Exposed (>20–43%) vs. unexposed (0%)* 0% vs. 7.5% OR: unable to calculate	*Exposed (>20–43%) vs. unexposed (0%)* aOR^‡^: unable to calculate *P* = 0.03
Roh et al.^24^	565 Pregnant women living with HIV	185 (32.7) *>0–90% of pregnancy:* 88 (15.6)	380 (67.3)	*Exposed (>0–90%) vs. unexposed (0%)* 5.7% vs. 5.3% RR: 1.08 (0.42–2.80)	*Exposed (>0–90%) vs. unexposed (0%)* aRR^§^: 0.78 (0.22–2.72)
		*>90% of pregnancy:* 97 (17.2)		*Exposed (>90%) vs. unexposed (0%)* 2.1% vs. 5.3% RR: 0.39 (0.09–1.65)	*Exposed (>90%) vs. unexposed (0%)* aRR^§^: 0.24 (0.04–1.52)
Roh et al.^22^	2,045 Singleton stillbirths	Not reported	Not reported	Not reported	*Exposed vs. unexposed* IRR^‖^: 0.94 (0.50–1.77) First year after IRS start IRR: 1.02 (0.55–1.89) Second year after IRS start IRR: 0.87 (0.39–1.91)
Birth Weight (mean)					
Tongo et al.^30^	796 Pregnant women at delivery	569 (71.5)	227 (28.5)	3.22 kg vs. 2.97 kg *P* = 0.13	Not reported
Muhindo et al.^23^	289 Pregnant women	155 (53.6) *>0–20% of pregnancy:* 90 (31.1)	134 (46.3)	Not reported	*Exposed (>0–20%) vs. unexposed (0%)* adjusted mean difference in birth weight**: 196 g (51–340 g)
		*>20–43% of pregnancy:* 65 (22.5)			*Exposed (>20–43%) vs. unexposed (0%)* adjusted mean difference in birth weight**: 257 g (105–409 g)
Gestational Age at Delivery (mean)					
Tongo et al.^30^	796 Pregnant women at delivery	569 (71.5)	227 (28.5)	38.0 weeks vs. 37.7 weeks *P* = 0.21	Not reported
Congenital Anomalies					
Bornman et al.^27^	3,310 Mother–infant dyads	2,396 (72)	914 (27.6)	Any external urogenital birth defect: 11% vs. 10.2%	*Exposed vs. unexposed* any external urogenital birth defect: aOR^††^: 1.33 (1.04–1.72)

aOR = adjusted odds ratio; aRR = adjusted RR; IRR = incidence RR; IRS = indoor residual spraying; OR = odds ratio.

* Ratio is the inverse of that reported in the original manuscript to keep associations in the same direction.

^†^ Tongo et al.^30^ adjusted for use of any other malaria preventive strategy, including mosquito coils, window nets, bed nets, repellent creams, pyrimethamine use, intermittent preventive treatment (IPT), or traditional herbs. They also controlled for maternal age and parity.

^‡^ Muhindo et al.^23^ adjusted for gravidity, household wealth, presence of parasites at enrollment, gestational age study drugs started, and type of IPT use.

^§^ Roh et al.^24^ adjusted for education, household wealth, receipt of IPT, baseline CD4^+^ T cell count, baseline HIV viral load, maternal age, reported bed net ownership, protease inhibitor use, and gravidity.

^‖^ Roh et al.^22^ used two approaches to determine the IRR, a machine learning approach (reported here) and a difference-in-difference approach. The effect size and direction were not meaningfully different between the approaches, so only the first is included here.

^¶^ Uwimana et al.^25^ adjusted for study arm, gravidity, baseline hemoglobin level, and treatment of malaria during the pregnancy before enrollment. The model for low birthweight was additionally adjusted for number of antenatal care visits.

^#^ Roh et al.^24^ and Roh et al.^22^ define fetal demise/stillbirth as delivery of a nonviable fetus ≥28 weeks gestational age. Muhindo et al.^23^ define fetal demise/stillbirth as including spontaneous abortion and stillbirth without specification of gestational age.

** Muhindo et al.^23^ adjusted for gravidity, gestational age when study drugs were started, wealth category, loop-mediated isothermal amplification at enrollment, and treatment arm.

^††^ Bornman et al.^27^ controlled for time residing in the village and maternal occupation.

**Figure 4.**
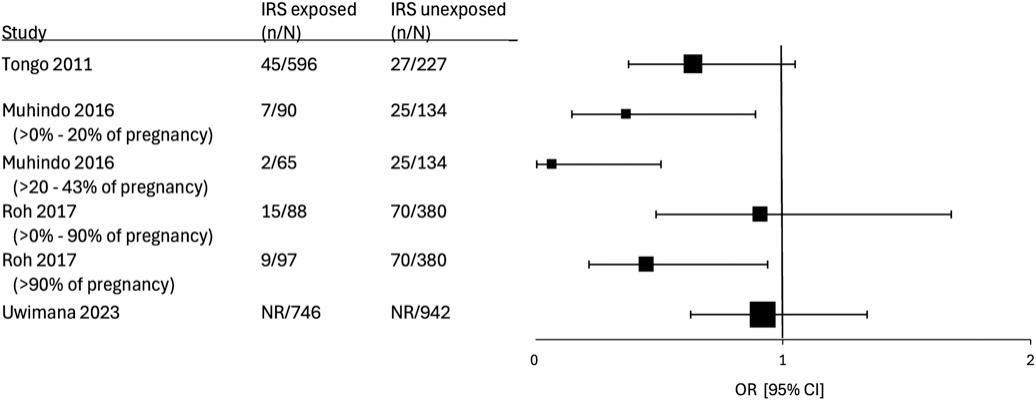
Forest plot of studies comparing low birthweight between those exposed and those not exposed to indoor residual spraying (IRS). ^†^Study used an unadjusted odds ratio (OR); ^‡^study used adjusted OR; and ^&^study used adjusted RR or incidence RR.

Five studies reported the rate of LBW, and two of these also reported on birth weight as a continuous variable. Muhindo et al.^23^ and Roh et al.^24^ both found reductions in LBW, which were greater the longer the pregnancy was protected by IRS (Figure 4). In 2022, Roh et al. performed a multivariate modeling approach on outcomes from almost 60,000 deliveries that confirmed this finding. Greater reductions in the rate of LBW were observed the longer an area was protected by IRS (first year after IRS start, IRR of 0.72 [95% CI 0.50–1.03], versus the second year after IRS start, IRR of 0.62 [95% CI 0.42–0.92]).^22^ Again, only Uwimana et al.^25^ was an outlier, showing no significant difference in LBW in those exposed to IRS (aRR: 1.04 [95% CI 0.69–1.56]).

Three studies reported on the incidence of fetal or neonatal demise, with mixed results. Muhindo et al.^23^ observed a substantial reduction in fetal/neonatal mortality regardless of the length of time of exposure to IRS (exposed >0–20% aRR of 0.10 [95% CI 0.0–0.86] versus exposed >20–43% aRR of 0.0 [95% CI not reported]; Table 3). Although Roh et al.^24^ found lower risk of fetal/neonatal mortality in their adjusted model, these results were not significant (exposed >0–90% aRR of 0.78 [95% CI 0.22–2.72] versus exposed >90% aRR of 0.24 [95% CI 0.04–1.52]). The follow-up study published by Roh et al.^22^ in 2022 did not show a significant reduction in rate of fetal or neonatal demise.

Finally, one study (Bornman et al.^27^) evaluated the risk of external urogenital birth defects in male infants born to mothers exposed to IRS. In an adjusted model, the risk of any urogenital birth defect was 33% greater in those exposed to IRS (aRR: 1.33 [95% CI 1.04–1.72]; Table 3). The absolute incidence of urogenital defect in the exposed group was 11% compared with 10.2% in the unexposed group.

### Risk of bias in included studies.

Using the NIH Quality Assessment Tool for Observational Cohort and Cross-Sectional Studies, 5 studies were evaluated as “Good”, 10 as “Fair”, and 2 as “Poor” (Supplemental Appendix 2). The five studies rated “Good” (Bornman et al.,^27^ Muhindo et al.,^23^ Roh et al.,^24^ Roh et al.,^22^ and Uwimana et al.^25^) met the quality assessment criteria in all but one or two categories. Not coincidentally, four of these five studies were prospective observational studies where the data were collected as part of an randomized controlled trial. The studies evaluated as “Fair” (Hamer et al.,^26^ Tongo et al.,^30^ Nega et al.,^31^ Kamuliwo et al.,^32^ Tilahun et al.,^33^ Subussu et al.,^34^ Alhassan et al.,^35^ Gemechu et al.,^36^ Balcha et al.,^37^ and Eboumbou Moukoko et al.^38^) had deficiencies regarding our specific study question on IRS, although we acknowledge that the objective of many of these studies was not to evaluate IRS exposure in pregnancy. None of the 12 studies evaluated as “Fair” or “Poor” reported the date of IRS spraying and so were unable to report whether the time frame from exposure to outcome was sufficient, nor were they able to examine different levels of exposure (e.g., length of pregnancy protected by IRS). In addition to the deficiencies noted above, the two studies judged as “Poor” (Lee et al.^28^ and Lee et al.^29^) do not report details on the population included. Both studies were cross-sectional series performed in the setting of other malaria control strategies, which are not accounted for in an adjusted effect size.

### Certainty of the body of evidence.

Based on most studies being rated as “Fair” or “Poor” quality by the NIH scale, the overall body of evidence evaluating the effect of IRS in pregnancy is fair. It is important to note that there are no prospective trials that have evaluated obstetric outcomes in the setting of IRS as their primary goal. Even among the five studies of “Good” quality in our review, three (Muhindo et al.,^23^ Roh et al.,^24^ and Uwimana et al.^25^) evaluate IRS as a secondary exposure, of which two evaluated a different intervention to prevent malaria (i.e., IPT). The largest and best-quality study to evaluate obstetric outcomes (Roh et al.^22^) was a quasiexperimental time series design that did not control for other malaria prevention measures that could have changed over time.^24^

## DISCUSSION

In this systematic review, we identified 17 studies that evaluated the risk of malaria and obstetric or neonatal outcomes in patients exposed to IRS during pregnancy. All the included studies were either observational or cross-sectional, with a substantial amount of methodologic variation between studies, and therefore the decision was made not to perform a meta-analysis of these results. Specifically, there were differences in how the exposure was defined (e.g., timing of IRS in relation to pregnancy, frequency of spraying, and type of insecticide used), whether other malaria prevention strategies were used, and differences in how outcomes were defined (e.g., malaria diagnosis was clinical or determined by a variety of laboratory techniques [RDT, blood smear, or PCR] with different sensitivities and specificities). Differences in effect estimates could also reflect the underlying degree of malaria endemicity or insecticide resistance among vectors in a geographic region. Despite this, there is a clear trend toward lower risk of malaria in pregnancy, as would be expected. Zhou et al.^9^ published a meta-analysis in 2022 that showed a 65% reduction in malaria incidence in those exposed to IRS across 38 studies (19 of which were in an adult population). Muhindo et al.^23^ saw a similar magnitude reduction in malaria of 58%. The results from several larger population-level studies included in our review (Lee et al.,^28^ Lee et al.,^29^ and Kamuliwo et al.^32^) corroborate the impact of IRS on malaria reduction.

We identified only five studies that examined obstetric outcomes in those exposed to IRS, none of which prospectively evaluated the impact of IRS as its primary exposure. The available evidence does indicate a lower rate of PTB and LBW in those exposed to IRS, although the degree of protection varies widely between studies, and one study (Uwimana et al.) indicates a higher rate of PTB and no change in the rate of LBW in those exposed to IRS.^22^^–^^25^^,^^30^ Encouragingly, two studies demonstrated a greater reduction in PTB and LBW the longer a pregnancy was exposed to IRS.^22^^,^^23^ Similarly, two prospective trials showed a reduction in fetal/neonatal mortality in those exposed to IRS, but a larger quasiexperimental study found no difference.^22^^–^^24^ Given the uncertainty in these findings, prospective studies that enroll individuals before pregnancy or early in pregnancy and follow them beyond delivery are needed to better define obstetric outcomes in relation to IRS. We did not identify any ongoing clinical trials assessing IRS exposure in relation to pregnancy outcomes.

One study showed a possible association between DDT and external urogenital defects in males. However, the rate of these anomalies is higher than would be expected in both groups, and the absolute increase in risk from 10.2% to 11% is clinically small.^27^ It is important to note that there is a larger body of literature exploring neonatal and early developmental outcomes in those exposed to pesticides. These were not included in this review as they used biomarker concentrations rather than IRS as the exposure of interest, and the concentrations likely reflect exposure from a variety of sources (e.g., diet, occupational agricultural pesticide use, residential pesticide use, and treatment of head lice) and as such cannot be linked solely to an IRS campaign. In the Venda Health Examination of Mothers, Babies and their Environment (VHEMBE) Study conducted in South Africa, pesticides frequently used in IRS were associated with abnormalities in neonatal thyroid hormones, early childhood growth, and neurocognitive development.^39^^–^^41^ Additionally, Prahl et al.^42^ demonstrated that prenatal exposure to bendiocarb, a carbamate insecticide, is associated with a variety of neonatal immune changes in the laboratory. These findings are reason to be cautious in supporting broader use of IRS in protecting pregnant persons based on the potential benefits summarized above and underscore a need for continued investment in developing alternate malaria control measures in pregnancy, such as vaccines.

In this review, we sought to understand what is known about the impact of IRS on malaria in pregnancy and other obstetric outcomes. There were six studies that reported on “insecticide sprays” that included self-reporting of personal spraying of insecticides.^15^^,^^17^^–^^20^ To reduce a potential bias in excluding these studies, we have included a summary of the outcomes (Supplemental Appendix 3). These studies similarly show a reduced risk of malaria in those exposed to “insecticide sprays” during pregnancy. Recently, an insecticide-treated durable wall lining has been developed to help reduce the potential exposure to insecticides that has a similar impact as IRS on mosquito populations.^43^ However, to date, there is no published study on its use in relation to pregnancy nor any published data on whether exposure levels of insecticide using this product is reduced compared with exposure following IRS campaigns.

## CONCLUSION

Despite the wealth of data on the efficacy of IRS in reducing malaria, there are limited published data on the specific effect of IRS on malaria in pregnancy or obstetric outcomes. IRS appears to protect pregnant women against parasitemia, reduce placental malaria, and may reduce rates of PTB, LBW, and fetal/neonatal mortality. However, without high-quality evidence to understand the potential risks and benefits of its use, no clear endorsement of its use to protect pregnant women can be made. Caution is also warranted given demonstrated negative long-term impact on childhood neurodevelopment from prenatal exposure to certain insecticides. The nature of an IRS campaign is to achieve population coverage, and therefore some pregnant individuals will invariable be exposed to insecticide. Increased investment in developing alternate malaria control measures in pregnancy, such as vaccines, is needed.

## Supplemental Materials

10.4269/ajtmh.24-0435 Supplemental Materials
