# Supplementary material for: Effect of Indoor Residual Spraying on Malaria in Pregnancy and Pregnancy Outcomes: A Systematic Review
Source: Am J Trop Med Hyg. 2024 Oct 29;112(2):253–65. doi: 10.4269/ajtmh.24-0435 (PMC11803663; doi:10.4269/ajtmh.24-0435)
Supplement: Supplemental Materials [file tpmd240435.SD1.pdf]

Appendix:

Appendix 1. Systematic review search terms

**Pubmed**	(Indoor residual spraying[tiab] OR IRS[tiab] OR insecticidetreated[tiab] OR ITWL[tiab] OR ITPS[tiab] OR ZeroFly[tiab] OR PermaNet[tiab] OR ZeroVector[tiab] OR (insecticid*[tiab] AND wall[tiab] AND liner[tiab])) AND ("Reproduction"[Mesh] OR "Pregnant Women"[Mesh] OR "Mothers"[Mesh] OR "Pregnancy"[Mesh] OR "Parturition"[Mesh] OR pregnan*[tiab] OR maternal[tiab] OR mother*[tiab] OR "malaria in pregnancy" OR "maternal Parasitemia") AND ("Pregnancy Complications, Parasitic"[Mesh] OR "Pregnancy Complications, Infectious"[Mesh] OR "Parasitemia"[Mesh] OR malaria*[tiab] OR parasitem*[tiab])
**Embase**	#1 'indoor residual spraying'/exp AND 'chemical pest control'/exp<br>#2 'indoor residual spraying':ab,ti OR irs:ab,ti OR 'insecticide treated':ab,ti OR itwl:ab,ti OR itps:ab,ti OR zerofly:ab,ti OR permanet:ab,ti OR zerovector:ab,ti<br>#3 insecticid*:ab,ti AND wall:ab,ti AND liner:ab,ti<br>#4 #1 OR #2 OR #3<br>#5 'reproduction'/exp OR 'pregnant woman'/exp OR 'mother'/exp OR 'pregnancy'/exp OR 'birth'/exp OR pregnan*:ab,ti OR maternal:ab,ti OR mother*:ab,ti OR 'malaria in pregnancy' OR 'maternal parasitemia':ab,ti<br>#6 'pregnancy disorder'/exp OR 'pregnancy complication'/exp OR 'parasitemia'/exp OR 'malaria'/exp OR malaria*:ab,ti OR parasitem*:ab,ti<br>#7 #5 AND #6
**Web of Science Core Collection (Clarivate)**	Editions (all available): SCI-EXPANDED, SSCI, A&HCI, CPCI-S, CPCI-SSH, BKCI-S, BKCI-SSH, ESCI, CCR-EXPANDED, IC.<br>TOPIC = ((Indoor residual spraying OR IRS OR insecticide-treated OR ITWL OR ITPS OR ZeroFly OR PermaNet OR ZeroVector OR (insecticid* AND (wall liner))))<br>AND<br>TOPIC = (("Reproduction" OR "Pregnant Women" OR "Mothers" OR "Pregnancy" OR "Parturition" OR pregnan* OR maternal OR mother* OR "malaria in pregnancy" OR "maternal Parasitemia") AND (Complication* OR Parasitemia OR malaria* OR parasit*)) ("Reproduction" OR "Pregnant Women" OR "Mothers" OR "Pregnancy" OR "Parturition" OR pregnan* OR maternal OR mother* OR "malaria in pregnancy" OR "maternal Parasitemia") AND (Complication* OR Parasitemia OR malaria* OR parasit*)
**Cochrane Central Register of Controlled Trials (Wiley, Cochrane Library)**	Searched in: title abstract keyword<br>Limits: search word variations<br>Indoor residual spraying

Appendix 2. Quality assessment of included studies[a]

	Was the research question or objective in this paper clearly stated?	Was the study population clearly specified and defined?	Was the participation rate of eligible persons at least 50%?	Were all the subjects selected or recruited from the same or similar populations (including the same time period)? Were inclusion and exclusion criteria for being in the study prespecified and applied uniformly to all participants?	Was a sample size justification, power description, or variance and effect estimates provided?	For the analyses in this paper, were the exposure(s) of interest measured prior to the outcome(s) being measured?	Was the timeframe sufficient so that one could reasonably expect to see an association between exposure and outcome if it existed?	For exposures that can vary in amount or level, did the study examine different levels of the exposure as related to the outcome (e.g., categories of exposure, or exposure measured as continuous variable)?	Were the exposure measures (independent variables) clearly defined, valid, reliable, and implemented consistently across all study participants?	Was the exposure(s) assessed more than once over time?	Were the outcome measures (dependent variables) clearly defined, valid, reliable, and implemented consistently across all study participants?	Were the outcome assessors blinded to the exposure status of participants?	Was loss to follow-up after baseline 20% or less?	Were key potential confounding variables measured and adjusted statistically for their impact on the relationship between exposure(s) and outcome(s)?	Quality Rating	Was IRS the primary intervention studied?
Hamer 2009	+	+	+	+	+	+	CD	-	+	-	+	NR	NA	-	Fair	No
Bornman 2010	+	+	NR	+	+	+	+	-	+	-	+	NR	NA	+	Good	Yes
Lee 2010	+	-	NR	-	+	-	CD	-	+	-	+	NR	NA	-	Poor	No
Lee 2010 (2)	+	-	NR	-	+	-	CD	-	+	-	+	NR	NA	-	Poor	No
Tongo 2011	+	+	+	+	+	-	CD	-	+	-	+	NR	NA	+	Fair	No
Nega 2015	+	+	+	+	+	+	CD	-	+	-	+	NR	NA	+	Fair	No
Kamuliwo 2015	+	+	NA	NA	+	NA	CD	-	+	+	+	NA	NA	-	Fair	No
Muhindo 2016	+	+	+	+	+	+	+	+	+	-	+	+	+	+	Good	Yes
Roh 2017	+	+	+	+	+	+	+	+	+	NR	+	+	+	+	Good	Yes
Tilahun 2020	+	+	NR	+	+	+	CD	-	+	-	+	NR	NA	+	Fair	No
Subussa 2021	+	+	+	+	+	+	CD	-	+	-	+	NR	NA	+	Fair	No
Alhassan 2022	+	+	+	+	+	-	CD	-	+	-	+	NA	NA	-	Fair	Yes
Roh 2022	+	+	NA	+	+	+	+	+	+	NA	+	+	NA	+	Good	Yes
Uwimana 2023	+	+	+	+	+	+	CD	-	+	-	+	+	+	+	Good	No
Gemechu 2023	+	+	NR	+	+	+	CD	-	+	-	+	NR	NA	+	Fair	No
Balcha 2023	+	+	NR	+	+	+	CD	-	+	-	+	NR	NA	+	Fair	No
Eboumbou Moukoko 2023	+	+	+	+	+	+	CD	-	+	-	+	NR	NA	+	Fair	No

[a]The NIH Quality Assessment Tool for Observational Cohort and Cross-Sectional Studies was utilized by two independent reviewers to evaluate the quality of included studies.

Appendix 3. Malaria outcomes of studies reporting on personal “insecticide sprays” rather than systematically sprayed IRS

**Peripheral parasitemia (asymptomatic)**					
**Study**	**Sample Size/Details**<br>**N**	**Insecticide spray exposed n (%)**	**Insecticide spray unexposed**<br>**n (%)**	**Effect size**<br>**Insecticide spray exposed vs unexposed**<br>**Crude ratio (95% CI)**	**Effect size**<br>**Adjusted ratio (95% CI)**
Agomo 2013	1,084 pregnant women, asymptomatic	885 (81.6%)	199 (18.4%)	OR: 0.36(0.24 – 0.54)	*Exposed vs unexposed*<br>aOR[a]: 0.37 (0.23 – 0.59)
Efunshile 2011	400 pregnant women, asymptomatic	131 (32.8%)	269 (67.3%)	2.3% vs 7.8%<br>OR: 0.28 (0.08 – 0.93)	Not reported
Obieche 2015	344 women within 24 hrs of delivery	11 (3.2%)	333 (96.8%)	*Among regular users of indoor insecticide sprays:*<br>9 (81.8%), no referent	Not reported
Maduakor 2019	548 pregnant women, asymptomatic	384 (70.1%)	164 (29.9%)	39.6% vs 86.6%<br>OR: 0.10 (0.06 – 0.17)	Not reported
Oladosu 2023	284 pregnant women, asymptomatic	91 (32.3%)	192 (67.8%)	15.4% vs 20.3%<br>OR: 0.71 (0.37 – 1.39)	Not reported
Dwumfour 2023	140 pregnant women, asymptomatic	61 (43.6%)	79 (56.4%)	OR: 0.38 (0.15 – 0.97)	*Exposed vs unexposed*<br>aOR[a]: 0.27 (0.09 – 0.84)

[a] Agomo 2013 and Dwumfour 2023 did not report the variables used in the multivariate model.

Appendix 4: Characteristics of included studies (Ordered by publication year)

Burden of malaria in pregnancy in Jharkhand State, India
Hamer 2009

**Methods**	**Aim**: To define the burden of malaria in pregnancy in a malaria-endemic state in central-east India<br>**Study Design**: Facility-based cross-sectional study<br>**Dates:** December 2006 – December 2007
**Participants**	**Number of participants**: 3,104 total<br>• 2,386 pregnant women enrolled at antenatal clinics<br>• 718 enrolled at time of delivery<br>**Population Characteristics:**<br>• Pregnant women aged >15 years enrolled either during antenatal care or at time of delivery
**Location**	**Study Location**: Jharkhand State, India<br>**Malaria Endemicity**: Year-round endemic
**IRS Details**	**Definition of IRS exposure:** Self-report of IRS exposure to home (questionnaire), dichotomous (yes/no)<br>**IRS specifics:**<br>• Typical insecticide used in area by government at time either dichloro-diphenyl-trichlo-roethane (DDT) (Ranchi district) or synthetic pyrethroids (Gumla district)<br>• Timing of IRS exposure in relation to pregnancy not reported
**Outcomes evaluated**	**Malaria outcomes:** Peripheral parasitemia (microscopy or RDT), placental parasitemia, and maternal anemia<br>**Obstetric outcomes:** None in relation to IRS
**Notes**	• Birth outcomes (low birthweight, preterm birth, stillbirth and gestational hypertension) reported by presence of placental parasitemia but not by IRS exposure<br>• Other malaria prevention practices: ITNs, IPT

DDT and urogenital malformations in newborn boys in a malarial area
Bornman 2010

**Methods**	**Aim**: Determine the association of external urogenital birth defects in newborn boys with DDT exposure from spraying in a malaria area<br>**Study Design**: Facility-based cross-sectional study<br>**Dates:** May 2004 – May 2006
**Participants**	**Number of participants**: 3310 mother-newborn dyads<br>**Population Characteristics:**<br>• Full-term newborn males born in Tshilidzini Hospital

	• Mothers residing outside of Limpopo Province, who were private patients (not public patients), and babies with birth mass of <2.5kg were excluded
**Location**	**Study Location**: Vhembe District, Limpopo, South Africa<br>**Malaria Endemicity**: High endemicity
**IRS Details**	**Definition of IRS exposure:** Binary exposure determined on a village level by information provided by Limpopo Malaria Control Program<br>**IRS specifics:** DDT sprayed annually by Limpopo Malaria Control Program since 1966
**Outcomes evaluated**	**Malaria outcomes:** None<br>**Obstetric outcomes:**<br>• External urogenital birth defects in male infants diagnosed at birth
**Notes**	• Other malaria prevention practices: None

Pre-elimination of malaria on the island of Principe
Lee 2010

**Methods**	**Aim**: To evaluate the impact of an integrated malaria control program on the prevalence and epidemiology of malaria in Principe<br>**Study Design**: Cross-sectional<br>**Dates:** July 2009
**Participants**	**Number of participants**: 5,609 total<br>**Population Characteristics:** Inhabitants of Principe<br>• Number of pregnant participants is not reported<br>• Inclusion and exclusion criteria are not defined. Participation rate is not reported.
**Location**	**Study Location**: Principe<br>**Malaria Endemicity**: Year-round highly endemic prior to program (prevalence of parasitemia 35% in 1997)
**IRS Details**	**Definition of IRS Exposure:** Dichotomous (yes/no)<br>**IRS Specifics:** alphacypermethrin (pyrethroid insecticide), sprayed yearly starting in 2003
**Outcomes evaluated**	**Malaria outcomes:** Incidence of malaria in pregnant participants<br>• Though the overall effect of IRS on malaria incidence is reported, it is not reported among pregnant individuals alone.<br>**Obstetric outcomes:** None
**Results**	**Malaria outcomes:** Absolute incidence of malaria in pregnancy
**Notes**	IRS not only malaria control intervention utilized in this time period and therefore reduction cannot only be attributed to IRS; other interventions included scaling of IPT, LLINs, and larviciding<br>Unclear if active surveillance cross-sectional surveys included information on pregnancy or whether incidence in pregnancy calculated from passive surveillance only

Potential threat of malaria epidemics in a low transmission area, as exemplified by Sao Tome and Principe
Lee 2010 (2)

**Methods**	**Aim**: To evaluate the impact of an integrated malaria control program on the prevalence and epidemiology of malaria in Sao Tome and Principe<br>**Study Design**: Cross sectional<br>**Dates:** July 2009
**Participants**	**Number of participants**: 12,463 inhabitants<br>**Population Characteristics:** Inhabitants living at malaria transmission hotspots (located at Agua Grande and Me-Zoxi)
**Location**	**Study Location**: Sao Tome and Principe<br>**Malaria Endemicity**: Year-round highly endemic prior to program (prevalence of parasitemia 35% in 1997)
**IRS Details**	**Definition of IRS Exposure:** Dichotomous (yes/no)<br>**IRS Specifics:** alphacypermethrin (pyrethroid insecticide), sprayed yearly across entire island starting in 2004
**Outcomes evaluated**	**Malaria outcomes:** Proportion of pregnant women with malaria diagnostic that is slide positive for *P. Falciparum*<br>**Obstetric outcomes:** None
**Notes**	IRS not only malaria control intervention utilized in this time period and therefore reduction cannot only be attributed to IRS; other interventions included scaling of IPT, LLINs, and larviciding

Utilisation of malaria preventive measures during pregnancy and birth outcomes in Ibadan, Nigeria
Tongo 2011

**Methods**	**Primary Study Aim**: To determine association between malaria preventive measures utilized during pregnancy and birth outcomes<br>**Study Design**: Facility-based cross-sectional study<br>**Dates:** November 2007 - January 2008
**Participants**	**Number of participants**: 796 pregnant women<br>**Population characteristics:** Pregnant women delivering at either University College Hospital or Adeoyo Maternity Hospital<br>• Recruited consecutively during weekdays at time of delivery<br>• Interviewed within 24 hours of birth
**Location**	**Study location**: Ibadan, Nigeria<br>**Malaria endemicity**: Year-round, highly endemic
**IRS details**	**Definition of IRS exposure:** self-report (questionnaire) as exposed in pregnancy to ‘insecticide space spray’ Dichotomous (yes/no)<br>**Insecticide specifics:** not described<br>**Other MIP prevention interventions assessed**: ITN, IPT, window nets, insecticide creams, mosquito coils

**Outcomes evaluated**	**Malaria outcomes:** None<br>**Obstetric outcomes:**<br>• Gestational age at delivery determined by LMP, US or Ballard examination<br>• Birth weight to nearest 50g with digital scales<br>• Preterm birth, <37 weeks at delivery<br>• Low birth weight, <2.5 kg
**Notes**	• Unclear whether insecticide space sprays represent community deployed IRS vs personal spraying of homes<br>• Other malaria prevention practices: Window nets, Mosquito coils, Bed nets, Weekly or intermittent treatment, Traditional herbs, LLINs, Mosquito repellent creams

Prevalence and predictors of asymptomatic malaria parasitemia among pregnant women in the rural surroundings of Arbaminch Town, South Ethiopia
Nega 2015

**Methods**	**Aim**: To determine the prevalence and predictors of asymptomatic *Plasmodium* infection among pregnant women in the rural District surrounding Arbaminch Town, Southern Ethiopia<br>**Study Design**: Community-based cross-sectional study<br>**Dates:** April to June 2013
**Participants**	**Number of participants**: 341 pregnant women<br>**Population Characteristics:**<br>• Pregnant women with absence of disease symptom/sign within the past 48 hours, axillary temperature ≤ 37.5°c, permanent residents in the study area, and those willing to participate in the study were included.<br>• Individuals having taken anti-malarial drugs in the past six weeks prior to data collection, or those undergoing any kind of long-term medical treatments were excluded.<br>• Recruited from community; villages and households selected by simple random sampling after identification of households with pregnant individuals by community health workers
**Location**	**Study Location**: Rural Surroundings of Arbaminch Town, Ethiopia<br>**Malaria Endemicity**: Year-round unstable transmission
**IRS Details**	**Definition of IRS exposure:** Self-report of IRS exposure to home within past 12 months (questionnaire), dichotomous (yes/no)<br>**IRS Specifics:** not reported
**Outcomes evaluated**	**Malaria outcomes:** Asymptomatic peripheral parasitemia (*Plasmodium)* determined by microscopy and RDT<br>**Obstetric outcomes:** None
**Notes**	• Other malaria prevention practices: ITNs

Spatial patterns and determinants of malaria infection during pregnancy in Zambia
Kamuliwo 2015

**Methods**	**Aim**: To determine the burden of and risk factors for malaria in pregnancy<br>**Study Design**: Cross-sectional<br>**Dates:** January 2009 to December 2014
**Participants**	**Number of participants**: Not specified<br>**Population Characteristics:**<br>• Monthly district-level, aggregated malaria cases (count data) from pregnant women from Zambian District Health Information System (DHIS). DHIS data were collected from all health facilities and kept in an electronic database
**Location**	**Study Location**: Zambia<br>**Malaria Endemicity**: Three zones: stable transmission (southeastern), unstable transmission (northeastern, northwestern), low transmission (southwestern)
**IRS Details**	**Definition of IRS exposure:**<br>• District level aggregated data on IRS coverage were collected from spray forms kept by the National Malaria Control Center (NMCC) in Zambia<br>• Exposure defined as total number of houses sprayed in a district per year<br>**IRS Specifics:** Not reported
**Outcomes evaluated**	**Malaria outcomes:** Prevalence rate ratio of malaria in pregnancy<br>**Obstetric Outcomes:** None
**Notes**	• Other malaria prevention practices: LLINs, IPTp

Reductions in malaria in pregnancy and adverse birth outcomes following indoor residual spraying of insecticide in Uganda
Muhindo 2016

**Methods**	**Primary Study Aim**: To assess the impact of IRS on malaria in pregnancy and birth outcomes<br>**Study Design**: Prospective cohort constructed within RCT of IPTp[1]<br>**Dates:** June 2014 – February 2015
**Participants**	**Number of participants**: 289 pregnant women<br>**Population Characteristics:**<br>• HIV-uninfected pregnant women<br>• ≥ 16 years of age of all gravidities<br>• Enrolled into RCT comparing IPT with dihydroartemisinin-piperaquine with IPT-sulfadoxine-pyrimethamine with information through delivery available
**Location**	**Study Location**: Tororo, Uganda<br>**Malaria Endemicity**: Year-round highly endemic
**IRS details**	**Definition of IRS Exposure:** Exposure to IRS if house or surrounding village sprayed, as determined by home visitors who determined exact date of spraying<br>• Considered fully exposed 14 days after spraying, to account for *P. falciparum* incubation<br>• 3 categories of IRS exposure:

	○ No protection: 0% of pregnancy exposed to IRS<br>○ >0 to 20% of pregnancy exposed<br>○ >20% to 43% of pregnancy exposed<br>• Second categorization of exposure<br>○ Direct (home sprayed)<br>○ Indirect (surrounding village sprayed)<br>○ None<br>**IRS Specifics:** Bendiocarb (carbamate insecticide), introduced by government midway through parent trial<br>**Other MIP prevention interventions utilized**:<br>• All trial participants given long-lasting insecticide treated bed net<br>• Received IPTp with either SP or DP
**Outcomes evaluated**	**Malaria outcomes:**<br>• Peripheral parasitemia in pregnancy by LAMP<br>• Placental malaria, by LAMP and histopathology<br>**Obstetric Outcomes*:**<br>• Birth weight<br>• Low birth weight<br>• Preterm delivery (< 37 weeks)<br>• Fetal/neonatal death (spontaneous abortion or stillbirth or neonatal death within 4 weeks)<br>**For twins, adverse outcome counted if present in either twin.*
**Notes**	• Other malaria prevention practices: ITNs, IPTp (primary intervention in the original trial)

Protective Effect of Indoor Residual Spraying of Insecticide on Preterm Birth Among Pregnant Women with HIV Infection in Uganda: A Secondary Data Analysis
Roh 2017

**Methods**	**Primary Study Aim**: To determine association of IRS with birth outcomes in pregnant women living with HIV<br>**Study Design**: Prospective cohort within a randomized controlled trial<br>• Secondary data analysis from two prospective trials enrolling pregnant women living with HIV; one conducted prior to IRS campaign (PROMOTE-PIs, NCT00993031) and a second conducted after IRS campaign (PROMOTE-BC2, NCT02282293)<br>**Dates:** Pre/post IRS campaign in 2014<br>• PROMOTE-PIs: December 2009 – July 2013<br>• PROMOTE-BC2: December 2014 – May 2016
**Participants**	**Number of participants**: 565 pregnant women<br>**Population Characteristics:** Pregnant women living with HIV enrolled into either PROMOTE-PIs or PROMOTE-BC2<br>• ≥ 16 years of age

	• Carrying singletons<br>• Data available through delivery
**Location**	**Study Location**: Tororo, Uganda<br>**Malaria Endemicity**: Year-round highly endemic
**IRS details**	**Definition of IRS exposure:** Exposure to IRS if house or adjacent home sprayed; timing in relation to pregnancy determined<br>• Considered fully exposed 14 days after spraying, to account for *P. falciparum* incubation<br>• 3 categories of IRS exposure duration:<br>0% of pregnancy exposed to IRS<br>>0 to 90% of pregnancy exposed<br>>90% of pregnancy exposed<br>• Collapsed to binary variable (exposed, > 0% vs unexposed 0%) when outcomes rare<br>**Insecticide specifics:** Bendiocarb (carbamate insecticide)<br>• District wide IRS q 6 months, starting December 2014
**Outcomes evaluated**	**Malaria outcomes:**<br>• Symptomatic malaria in pregnancy, defined as fever plus positive blood smear<br>• Placental malaria, determined by combination of microscopy, LAMP/PCR, and histopathology<br>**Obstetric outcomes:**<br>• Preterm birth, <37 weeks at delivery<br>• Low birth weight, <2500 grams<br>• Fetal/neonatal death, composite of SAB <28 wks OR stillbirth $\geq$ 28 wks OR neonatal death within 28 days of birth
**Notes**	• Other MIP prevention interventions utilized: All women received combination ART, daily TMP-SMX prophylaxis (Primary outcome of the original trial), IPT, and ITNs

Prevalence of asymptomatic Plasmodium species infection and associated factors among pregnant women attending antenatal care at Fendeka town health facilities, Jawi District, North west Ethiopia: A cross-sectional study
Tilahun 2020

**Methods**	**Aim**: To assess the prevalence of asymptomatic *Plasmodium* infections in pregnancy and associated risk factors<br>**Study Design**: Facility-based cross-sectional study<br>**Dates:** February 2019 – March 2019
**Participants**	**Number of participants**: 331 pregnant women<br>**Population Characteristics:**<br>• Asymptomatic pregnant women<br>• Recruited from two antenatal care clinics with convenience sampling
**Location**	**Study Location**: Jawi District (northwest) Ethiopia

	**Malaria Endemicity**: Year-round endemic
**IRS details**	**Definition of IRS exposure:** Self-report of IRS exposure within past year, dichotomous (yes/no)<br>**IRS specifics:** not described<br>**Other interventions utilized:** ITNs
**Outcomes evaluated**	**Malaria outcomes:** Peripheral parasitemia by microscopy and RDT<br>**Obstetric outcomes:** None
**Notes**	• Unclear whether parasitemia was positivity by either microscopy or RDT<br>• Other malaria prevention practices: ITNs

Asymptomatic Plasmodium infection and associated factors among pregnant women in the Merti district, Oromia, Ethiopia
Subussa 2021

**Methods**	**Aim**: To determine the prevalence of asymptomatic *Plasmodium* parasitemia and associated factors among pregnant women.<br>**Study Design**: Community-based cross-sectional study<br>**Dates:** March – September 2018
**Participants**	**Number of participants**: 364 pregnant women<br>**Population Characteristics:**<br>• Pregnant women living in district during study period<br>• Included if asymptomatic. Excluded if received antimalarial medications in prior two weeks
**Location**	**Study Location**: Merti district, Oromia, Ethiopia<br>**Malaria Endemicity**: Year-round endemic
**IRS Details**	**Definition of IRS exposure**: Self-report of IRS exposure to home, dichotomous (yes/no)<br>**IRS Specifics:** Not described<br>**Other interventions utilized**: ITNs
**Outcomes evaluated**	**Malaria outcomes:** Peripheral parasitemia by microscopy or RDT<br>**Obstetric outcomes:** None
**Notes**	• Unclear whether parasitemia was positivity by either microscopy or RDT<br>• Other malaria prevention practices: ITNs

Impact of insecticide-treated nets and indoor residual spraying on self-reported malaria prevalence among women of reproductive age in Ghana: implication for malaria control and elimination
Alhassan 2022

**Methods**	**Aim**: To quantify the impact of household access to insecticide-treated nets (ITNs) and indoor residual spraying (IRS) on self-reported malaria prevalence among women of reproductive age<br>**Study Design**: Cross-sectional<br>**Dates:** October – December 2016
**Participants**	**Number of participants**: 4861 Total (350 pregnant women)

	**Population Characteristics:**<br>• Utilized data from nationwide Ghana Malaria Indicator Survey<br>• Women of reproductive age (15-49 years old) with complete data<br>• Abstracted data for this review only for those women who were currently pregnant
**Location**	**Study Location**: Ghana<br>**Malaria Endemicity**: Year-round endemic
**IRS details**	**Definition of IRS exposure:** Self-report of IRS exposure to home, within 12 months, dichotomous (yes/no)<br>**IRS specifics:** Not described<br>**Other interventions utilized:** ITNs
**Outcomes evaluated**	**Malaria outcomes:** Self-reported malaria episode within 12 months of interview<br>**Obstetric outcomes:** None
**Notes**	• Other malaria prevention practices: ITNs

Association between indoor residual spraying and pregnancy outcomes: a quasi-experimental study from Uganda
Roh 2022

**Methods**	**Aim**: To quantify the impact of a large-scale IRS campaign on pregnancy outcomes in Eastern Uganda<br>**Study Design**: Retrospective quasi-experimental Difference-in-Difference study<br>**Dates:** January 2013 -- May 2017
**Participants**	**Number of participants**: Data abstracted from 59,992 deliveries<br>**Population Characteristics:**<br>• Birth records obtained from routine surveillance data at 25 health facilities from five districts that were part of the IRS campaign and six neighboring control districts<br>• Three Health facilities were randomly selected from each district. Facilities were excluded if they averaged <200 births per year, were <5 km away from a neighboring district (to mitigate treatment misclassification), had low-quality data (defined as either missing complete months of data for >25 months during study period or missing covariates and/or outcome data for >30% of records).<br>• Individual-level birth records collected from all singleton deliveries
**Location**	**Study Location**: Eastern Uganda<br>**Malaria Endemicity**: Year-round highly endemic
**IRS details**	**Definition of IRS exposure:** Living in a district post-IRS campaign (exposed) vs living in control district (unexposed) or IRS targeted district pre-campaign (unexposed)<br>• IRS coverage of homes during campaigns reported at $\geq$ 92%<br>**IRS specifics:**<br>• Bendiocarb (carbamate insecticide), applied biannually from 2013 to 2015

	• Actellic 300CSVR (organophosphate insecticide) replaced bendiocarb in 2016-17, reapplied annually
**Outcomes evaluated**	**Malaria outcomes:** None<br>**Obstetric outcomes:** Incidence of low birthweight (<2500 grams, liveborn) and stillbirth<br>• Birth records abstracted from routine surveillance data at selected health facilities at individual level and aggregated to number of LBW or stillbirth per month per facility<br>• Compared pre/post difference in birth outcomes among IRS exposed vs unexposed (difference in differences) (first approach to analyses)<br>• Alternate analytic technique (matrix completion with nuclear norm minimization)<br>• Subgroup analyses by HIV and gravidity (primigravid/multigravida)<br>• Did not distinguish between antepartum and intrapartum stillbirth
**Notes**	• Other malaria prevention practices: None reported

Effectiveness of Intermittent Screening and Treatment of Malaria in Pregnancy on Maternal and Birth Outcomes in Selected Districts in Rwanda: A Cluster Randomized Controlled Trial
Uwimana 2023

**Methods**	**Aim**: To assess the impact of intermittent screening and treatment of malaria in pregnancy (ISTp) compared with routine care (screening for fever and testing only febrile women) on women's risk for placental and peripheral malaria at the time of delivery<br>**Study Design**: Prospective cohort within a cluster randomized controlled trial<br>**Dates:** September 2016 – June 2018
**Participants**	**Number of participants**: 1786 pregnant women<br>**Population Characteristics:**<br>• Pregnant women presenting for their first ANC visit were consecutively enrolled.<br>• Eligibility criteria included age ≥18 years, residence in the study area, willingness to have a supervised delivery, and providing informed consent
**Location**	**Study Location**: Kamonyi and Huye districts, Southern Province, Rwanda<br>**Malaria Endemicity**: High endemicity
**IRS details**	**Definition of IRS exposure:** Dichotomous (yes/no)<br>**IRS specifics:** IRS with a carbamate insecticide was conducted in April 2017 in Huye, but not Kamonyi District, with coverage of 96.6%
**Outcomes evaluated**	**Malaria outcomes:**<br>• Malaria infection by positive blood smear or RDT<br>• Placental malaria<br>• Maternal anemia was classified as any (Hb <11 g/dL) or moderate to severe (Hb <10 g/dL)<br>**Obstetric outcomes:**

	• Prematurity was defined as delivery <37 weeks' gestation, and LBW as <2500 g
**Notes**	• Other malaria prevention practices: ITNs (provided to all at beginning of trial), Intermittent screening and treatment of malaria in pregnancy (primary outcome of trial)

Asymptomatic Malaria During Pregnancy: Prevalence, Influence on Anemia and Associated Factors in West Guji Zone, Ethiopia – A Community Based Study
Gemechu 2023

**Methods**	**Aim**: To determine the prevalence of asymptomatic malaria and associated risk factors among pregnant women<br>**Study Design**: Community-based cross-sectional study<br>**Dates:** February 2022 – March 2022
**Participants**	**Number of participants**: 557 pregnant women<br>**Population Characteristics:**<br>• All pregnant women who were permanent residents of the selected kebeles in the West Guji zone, had axillary temperature below 37°C, and had no symptoms or signs of malaria.<br>• Pregnant women who had taken antimalarial medication within the previous two weeks were excluded.
**Location**	**Study Location**: West Guji Zone, Oromia, southern Ethiopia<br>**Malaria Endemicity**:
**IRS details**	**Definition of IRS exposure:** Self-reported, dichotomous (yes/no)<br>**IRS specifics:** Not reported.<br>**Other interventions utilized**: ITN use, outdoor vector control, mosquito repellent
**Outcomes evaluated**	**Malaria outcomes:** Plasmodium infection by RDT and microscopy<br>**Obstetric outcomes:** None
**Notes**	• Other malaria prevention practices: Mosquito repellent, outdoor vector control, ITNs

Prevalence of asymptomatic malaria and associated factors among pregnant women at Boset District in East Shoa Zone, Oromia Region, Ethiopia: a cross-sectional study
Balcha 2023

**Methods**	**Aim**: To assess the prevalence of asymptomatic malaria and associated factors among pregnant women<br>**Study Design**: Community-based cross-sectional<br>**Dates:** February to March 2022
**Participants**	**Number of participants**: 328 pregnant women

	**Population Characteristics:**<br>• All pregnant women with the absence of disease symptoms/signs of malaria within the past 48 h, axillaries temperature ≤ 37.5 ℃, and permanent residents in the study area.<br>• Excluded if they had taken antimalarial drugs in the last 4 weeks
**Location**	**Study Location**: Boset District, Oromia Regional State, Ethiopia.<br>**Malaria Endemicity**:
**IRS details**	**Definition of IRS exposure:** Self-reported, dichotomous (yes/no)<br>**IRS specifics:** Not reported<br>**Other interventions utilized**: ITNs,
**Outcomes evaluated**	**Malaria outcomes:** Asymptomatic plasmodium infection by RDT and microscopy<br>**Obstetric outcomes:** None
**Notes**	• Other malaria prevention practices: ITNs

Effectiveness of Intermittent Preventive Treatment with Sulfadoxine-Pyrimethamine in Pregnancy: Low Coverage and High Prevalence of Plasmodium falciparum dhfr-dhps Quintuple Mutants as Major Challenges in Douala, an Urban Setting in Cameroon
Eboumbou Moukoko 2023

**Methods**	**Aim**: To determine IPTp-SP adherence and coverage, and the impact on maternal infection and birth outcomes in the context of widespread SP resistance<br>**Study Design**: Facility-based cross-sectional<br>**Dates:** December 2015 – December 2016
**Participants**	**Number of participants**: 888 pregnant women<br>**Population Characteristics:**<br>• Pregnant women attending the health facilities for antenatal care (ANC) visits and delivery.<br>• Women infected with HIV, under cotrimoxazole therapy, with a documented history of allergy to sulfamides and having recently taken SP alone or in combination with folic acid were excluded from the study.
**Location**	**Study Location**: Deido District Hospital, Bonassama District Hospital, Nylon St Paul Maternity Clinic, Douala, Littoral region, Cameroon<br>**Malaria Endemicity**: Highly endemic
**IRS details**	**Definition of IRS exposure:** Self-reported, dichotomous (yes/no)<br>**IRS specifics:** Not reported<br>**Other interventions utilized**: IPTp-SP, ITNs
**Outcomes evaluated**	**Malaria outcomes:** Plasmodium falciparum infection by qPCR<br>**Obstetric outcomes:** None
**Notes**	• Other malaria prevention practices: ITNs, IPTp-SP (Primary intervention of trial)
